# Supplementary material for: Reconciling Mining with the Conservation of Cave Biodiversity: A Quantitative Baseline to Help Establish Conservation Priorities
Source: PLoS One. 2016 Dec 20;11(12):e0168348. doi: 10.1371/journal.pone.0168348 (PMC5173368; doi:10.1371/journal.pone.0168348)
Supplement: S1 Dataset — (ZIP) [file pone.0168348.s002.zip › Taxa/Serra Norte/SN_2013/N4E/Lista N4E-23.pdf]

CAVIDADE N4E-0023

Classe	Ordem	Fam/Outros	Gên/Outros	Espécie	S-U	U-S	Zon.
Annelida	Oligochaeta			jovem		4	E/P/A
Arachnida	Acari	Ixodidae	*Ixodes*	sp.1		X	E
Arachnida	Acari	Labidostommatidae		sp.1		X	E
Arachnida	Acari	Mesostigmata		jovem	X		
Arachnida	Acari	Prostigmata		jovem	X		
Arachnida	Acari	Trombiculidae		sp.39		X	E/P/A
Arachnida	Araneae	Corinnidae	*Abapeba*	sp.1	1		
Arachnida	Araneae	Dipluridae		jovem	1		
Arachnida	Araneae	Ochyroceratidae	*Ochyrocera*	sp.1		X	E
Arachnida	Araneae	Ochyroceratidae	*Speocera*	sp.1	X	X	E
Arachnida	Araneae	Pholcidae		jovem	X	X	E
Arachnida	Araneae	Prodidomidae		jovem		X	E
Arachnida	Araneae	Salticidae		sp.	X		
Arachnida	Araneae	Scytodidae	*Scytodes*	*eleonorae*	X	1	E
Arachnida	Araneae	Theridiosomatidae	*Plato*	sp.1	X	X	E/P/A
Arachnida	Opiliones	Escadabiidae		sp.2	X		
Arachnida	Opiliones	Sclerosomatidae	Gagrellinae	sp.	X		
Arachnida	Palpigradi	Eukoeneniidae	*Eukoenenia*	sp.1		X	E
Arachnida	Pseudoscorpiones	Chernetidae	*Spelaeochernes*	sp.1	X	X	E/P/A
Arachnida	Pseudoscorpiones	Chthoniidae	*Pseudochthonius*	sp.1	X	X	E
Diplopoda	Glomeridesmida	Glomeridesmidae		sp.	X		
Diplopoda	Polydesmida	Chelodesmidae		sp.3		7	E/P
Diplopoda	Polydesmida	Pyrgodesmidae		sp.2	X	X	E/P/A
Entognatha	Collembola	Isotomidae		sp.1		X	E
Entognatha	Collembola	Paronellidae		sp.1		X	E
Entognatha	Collembola	Paronellidae		sp.4		X	E/P/A
Entognatha	Collembola	Sminthuroidea		sp.2	X	X	E/P/A
Entognatha	Diplura	Campodeidae		sp.1		X	E
Entognatha	Diplura	Projapygidae		sp.1		X	E
Insecta	Blattodea			jovem	X	6	E/P
Insecta	Coleoptera	Carabidae		jovem	X		
Insecta	Coleoptera	Hydrophilidae		sp.10		X	P/A
Insecta	Coleoptera	Staphylinidae	Pselaphinae	sp.35		X	E/A
Insecta	Diptera	Cecidomyiidae		sp.	X		
Insecta	Diptera	Curtonotidae		sp.	X		
Insecta	Diptera	Dolichopodidae		sp.		X	A
Insecta	Diptera	Drosophilidae	*Drosophila*	jovem	X	X	P/A
Insecta	Diptera	Muscidae		sp.		X	P
Insecta	Diptera	Mycetophilidae		sp.	X		
Insecta	Diptera	Phoridae	Metopininae	sp.		X	E
Insecta	Diptera	Phoridae	Phorinae	sp.		X	A
Insecta	Diptera	Psychodidae	*Sciopemyia*	*sordellii*	X	X	E/P
Insecta	Diptera	Tipulidae	Tipulinae	sp.	X	X	E
Insecta	Heteroptera	Cydnidae		sp.	X	X	E/P
Insecta	Heteroptera	Dipsocoroidea		jovem		X	E
Insecta	Heteroptera	Reduviidae	*Zelurus*	sp.	X	2	E
Insecta	Hymenoptera	Formicidae	*Camponotus*	sp.	X		
Insecta	Hymenoptera	Formicidae	*Camponotus*	sp.1		X	E/A
Insecta	Hymenoptera	Formicidae	*Camponotus*	sp.2		X	P
Insecta	Hymenoptera	Formicidae	*Gnamptogenys*	sp.2		X	E
Insecta	Hymenoptera	Formicidae	*Hypoponera*	sp.1		X	E/P
Insecta	Hymenoptera	Formicidae	*Pachycondyla*	sp.1		X	E/P
Insecta	Hymenoptera	Formicidae	*Pachycondyla*	sp.2		X	E
Insecta	Hymenoptera	Formicidae	*Pseudomyrmex*	aff. *opaciceps*		X	E
Insecta	Hymenoptera	Formicidae	*Solenopsis*	sp.1	X	X	P

Insecta	Isoptera	Rhinotermitidae	*Heterotermes*	sp.		X	E
Insecta	Isoptera	Termitidae	*Nasutitermes*	sp.		X	E/P/A
Insecta	Isoptera	Termitidae		sp.	X		
Insecta	Lepidoptera	Tineoidea		jovem	X	X	P
Insecta	Orthoptera	Phalangopsidae	*Paraclodes*	jovem		1	E
Insecta	Orthoptera	Phalangopsidae	*Phalangopsis*	sp.	X	1	P
Insecta	Psocoptera	Psyllipsocidae	*Psyllipsocus*	sp.1		X	E
Insecta	Thysanura	Nicoletiidae		sp.1	X	X	E/P
Gastropoda	Pulmonata	Bulimulidae	*Naesiotus*	sp.		X	E/P
Gastropoda	Pulmonata	Subulinidae	*Lamellaxis*	sp.	X		
Gastropoda	Pulmonata	Subulinidae		sp.3		X	A
Gastropoda	Pulmonata	Systrophiidae	*Happia*	sp.		X	E
Malacostraca	Isopoda	Armadillidae		sp.1	X		
Malacostraca	Isopoda	Philosciidae		sp.1	X	X	E/P
Nematoda				sp.1		X	P/A
Amphibia	Anura			sp.		3	P/A
Mammalia	Chiroptera	Emballonuridae	*Peropteryx*	sp.	5		P/A
Mammalia	Chiroptera	Phyllostomidae	*Carollia*	*perspicillata*		28	P/A
Mammalia	Chiroptera	Phyllostomidae	*Trachops*	*cirrhosus*		1	P
Mammalia	Chiroptera	Phyllostomidae		sp.	30		P/A
